# Prevalence of iron deficiency in pregnant women: A prospective cross‐sectional Austrian study

**DOI:** 10.1002/fsn3.2588

**Published:** 2021-10-16

**Authors:** Harald Zeisler, Wolf Dietrich, Florian Heinzl, Philipp Klaritsch, Victoria Humpel, Manfred Moertl, Christian Obruca, Friedrich Wimazal, Angela Ramoni, Johanna Tiechl, Elisabeth Wentzel‐Schwarz

**Affiliations:** ^1^ Department of Obstetrics and Gynaecology Medical University Vienna Vienna Austria; ^2^ Department of Obstetrics and Gynaecology Karl Landsteiner University of Health Sciences University Hospital Tulln Tulln Austria; ^3^ Department of Obstetrics and Gynaecology Medical University Graz Graz Austria; ^4^ Department of Gynecology and Obstetrics Perinatal Center, Klagenfurt am Wörthersee Klagenfurt Austria; ^5^ Department of Obstetrics and Gynaecology Medical University Innsbruck Innsbruck Austria; ^6^ Department of Obstetrics St. Josef Krankenhaus Vienna Austria

**Keywords:** Austria, iron deficiency, maternal morbidity, pregnancy, prevalence, quality of life

## Abstract

The aim of the study was to determine, for the first time, in a prospective cross‐sectional multicenter study, the prevalence of iron deficiency (ID) in an Austrian pregnant population. A cohort of 425 pregnant women was classified into four groups of different weeks of gestation. Group 1 was monitored longitudinally, while groups 2–4, iron status, were sampled only once. Evaluation of the prevalence of ID was performed by comparing the diagnostic criteria of the WHO to the cutoff proposed by Achebe MM and Gafter‐Gvili A (Achebe) and the Austrian Nutrition Report (ANR). In comparison with the ANR, the prevalence of ID was lower in group 1 and higher in groups 2–4 (17.2% vs. 12.17%, 25.84%, 35.29%, and 41.76%, respectively) (*p*‐values < .01 except group 1). According to WHO, the prevalence in group 1 was 12.17% at inclusion, 2 months later 31.7%, and further 2 months later 65.71%, respectively. According to Achebe, the number of cases doubled; for group 1, the number of cases rose from 13 to 42 (115 patients total); for groups 2–4, we observed an increase from 112 to 230 (340 patients total). This study reported a prevalence of around 12% at the beginning of pregnancy, which increased during pregnancy up to 65%. ID can have a massive impact on quality of life, justifying screening, as iron deficiency would be easy to diagnose and treat.

## Graphical abstract

The aim of the study was to determine, for the first time, in a prospective cross‐sectional multicenter study the prevalence of iron deficiency (ID) in Austrian pregnant population. This study showed a prevalence of around 12% at the beginning of pregnancy, which increased during pregnancy up to 65%. ID can have a massive impact on the quality of life, so that screening would be entirely justified, as iron deficiency would be easy to diagnose and treat.

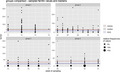

## 1 INTRODUCTION

Iron deficiency (ID) and iron deficiency anemia (IDA) are a global health problem affecting developing and developed countries. Pregnant women were identified as a risk group due to adverse outcome on pregnancy, maternal and fetal outcome (WHO and CDC, 2008). Pregnant women with IDA are at a higher risk of postpartum hemorrhage (PPH), receiving blood transfusion, and heart failure (Clevenger et al., 2016; Grewal, 2010; Kavle et al., 2008). In addition, ID and IDA during pregnancy can be considered as a risk factor for preterm delivery, low birth weight, perinatal, and neonatal mortality (Finkelstein et al., 2020; Georgieff, 2020; Rahman et al., 2016; Rahmati et al., 2019; Rao & Georgieff, 2007; Srour et al., 2018). Maternal iron deficiency, with or without associated anemia, has an adverse effect on fetal iron status because decreased maternal hemoglobin concentration is associated with decreased fetal iron stores (Means, 2020). Children with iron deficiency anemia show to have lower scores in cognitive, motor, social‐emotional, and neurophysiological development compared with group infants (Lozoff & Georgieff, 2006). Iron plays an important role in the growth and the development of the central nervous system and for the normal functioning of the brain (Lozoff et al., 2006). It is important for the obstetrician to know that human brain and cognitive development begin in the third trimester of pregnancy (Radlowski & Johnson, 2013). In Austria, screening for anemia, but not for iron deficiency, is part of the prenatal care (Mother‐Child‐Booklet). The ongoing discussions on a possible screening for iron deficiency in pregnancy always end with the reference to missing national data. Still now, there are neither data on the prevalence of iron deficiency nor on iron deficiency anemia in pregnancy in Austria. (Auerbach et al., 2021) reported that 42% of pregnant women were observed to be iron deficient in the first trimester. Stevens et al. reported a global prevalence of anemia, 2011, of nearly 30% of reproductive‐age women and 38% of pregnant women, respectively. The median prevalence in high‐income regions was 22% (16–29) for pregnant women aged 15–49 years (Stevens et al., 2013). Global anemia prevalence estimated by WHO with data from 1993 to 2005 revealed an estimated prevalence of IDA in pregnancy of 15.5% for Austria (WHO and CDC, 2008). In a recent study, 67% of Austrian pregnant women received iron supplementation, irrespective of whether they were deficient in iron with no information on the prevalence (Spary‐Kainz et al., 2019). Since iron deficiency is easy to diagnose and treat, the aim of this study was to evaluate the prevalence of iron deficiency in Austria for the first time. The results should be compared with the different diagnostic criteria for ID of the WHO (WHO and CDC, 2008) and cutoff proposed by Achebe MM and Gafter‐Gvili A (Achebe) (Achebe & Gafter‐Gvili, 2017) as well as to the Austrian Nutrition Report (ANR) 2012 (Elmadfa. 2012) that states that 17.2% of all nonpregnant women are affected by ID. The results should serve as the basis for the planned revised guideline of the Austrian Society for Obstetrics and Gynaecologists.

## 2 METHODS

### 2.1 Study participants and design

We conducted a cross‐sectional study in six Austrian hospitals between March 2017 and June 2020. The study participants were (singleton) pregnant women who were in obstetrical care at the Department of Obstetrics and Gynaecology, Medical University Vienna, Department of Obstetrics, St. Josef Krankenhaus, Vienna, Department of Gynaecology and Obstetrics, Perinatal Center Klagenfurt, Klagenfurt am Wörthersee, Department of Obstetrics and Gynaecology, Karl Landsteiner University of Health Sciences, University Hospital Tulln, Tulln an der Donau, Department of Obstetrics and Gynaecology, Medical University Innsbruck, Innsbruck, Department of Obstetrics and Gynaecology, Medical University Graz, Graz. The study participants were divided into four groups, depending on gestational age at inclusion: group 1: weeks 11 + 0 to 14 + 6, group 2: weeks 24 + 0 to 32 + 6, group 3: weeks 33 + 0 to 37 + 6, and group 4: weeks 38 + 0 to 41 + 6. Women in group 1 were monitored longitudinally to check for a change in iron status. In case of normal iron status, they were invited for a follow‐up visit 2 months after their first one, and then again, two months later for a third visit. For groups 2–4, iron status was sampled only once (at inclusion). In case of ID (A), iron supplementation was administered according to the algorithm suggested by Achebe and no further appointment was scheduled for patients from group 1. Medical history, pregnancy data, and laboratory parameters were taken from the medical record and collected in a web‐based database (eCRFs) by the company SCICOMED e.U. (www.scicomed.net) for all groups. For groups 2–4, we also collected diagnostic data (day of delivery, maternal and neonatal outcome) for possible further analysis. For early pseudonymization, each study participant received a unique identification number. Only the principal investigator of each participating center and their coworkers can link the identification number to the study participants.

### 2.2 Inclusion criteria

Signed informed consent, maternal age ≥18 and <46, singleton pregnancy, and weeks of gestation in accordance with the group definitions above.

### 2.3 Exclusion criteria

Ongoing iron supplementation at inclusion (except nutrient supplements), history of hemoglobinopathies or sickle cell anemia, gastrointestinal pre‐existing diseases (e.g., Crohn's Disease and Ulcerative Colitis), bariatric surgery, suspected bacterial and parasitic diseases (malaria, worm diseases, and Helicobacter pylori infection), and bleeding in case of placental disorders (placenta praevia, placenta accreta, increta, or percreta).

### 2.4 Parameters and definition of ID

The following parameters have been evaluated using the reference values of the Clinical Institute for Laboratory Medicine, General Hospital Vienna—Medical University Campus: hemoglobin 12.0–16.0 g/dl, ferritin 15–150 μg/l.

The prevalence of ID was examined with respect to three different definitions namely ANR, WHO, and Achebe. In ANR, the unusual cutoff of below 10 μg/l was used. With respect to WHO standards, ID is given by a ferritin level below 15 μg/l. Achebe and other studies warrant a serum ferritin level of <30 μg/l in pregnancy for the diagnosis of ID (Achebe & Gafter‐Gvili, 2017; DGHO, Leitlinie, 2018; Pavord et al., 2019; Bouri & Martin, 2018).

### 2.5 Statistics

Data are reported via median (numerical variables), respectively via absolute frequencies (categorical variables). Statistical tests were done with the R software package (version 4.0.3) (R Core Team, 2020). Data were plotted with the ggplot2 package (Wickham, 2016).

We employed a chi‐square‐goodnes of fit test to assess whether or not the results published in the Austrian Nutrition Report (ANR) 2012 (17.2% of all women show ID) hold true for pregnant women as well. Confidence intervals (95%) for the true value were computed via a two‐sample test for equality of proportions. The difference in parameters between groups was examined via Kruskal–Wallis tests, which were followed up by pair‐wise Wilcoxon rank‐sum tests in case of statistical significance. In order to investigate the correlation between gestational age (in weeks) and serum ferritin, we calculated Kendall's tau. Results with a *p*‐value less than .05 were considered statistically significant.

## 3 RESULTS

**FIGURE 1:**
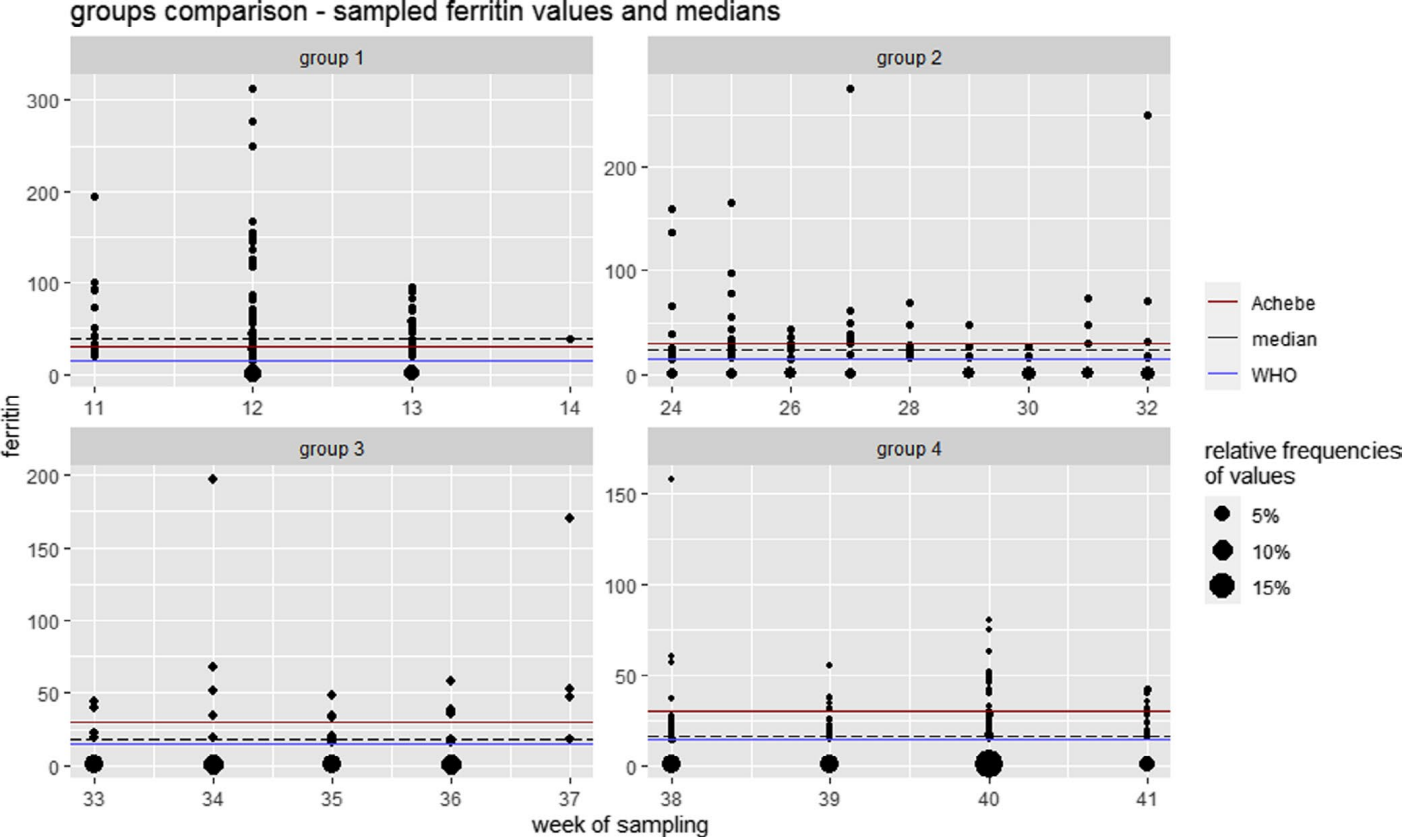
Scatter plot (gestational age in weeks at sampling/ferritin levels) detailing the distribution of ferritin levels in each group. Each dot corresponds to one observed value in the respective week and its size scales with the number of samples with the respective value. As such, the size reflects relative frequencies of the sampled values per week on a group level. For easy comparison, the median of the respective group (black dashed line) was included, as well as the two different cutoff points for the definition of ID (red = ÖGGG, blue = WHO). For better readability, different scales were used for each group

A total of 483 patients have been recruited across all sites; data from 425 women were used for analysis. Incomplete records were excluded. Patients have been divided into four groups as shown in Table 1. Table 2 represents selected data with respect to age, body mass index (BMI), and serum ferritin. We used the Kruskal–Wallis test to identify differences between the four groups. The results for age were not statistically significant; for BMI and serum ferritin, however, we computed *p*‐values < .01. Subsequent analysis via pair‐wise Wilcoxon rank‐sum tests revealed that patients from group 1 have lower prepregnancy BMI than the ones from the other groups (*p*‐values < .01; the other comparisons yielded no significant *p*‐values). For serum ferritin levels, we found a significant difference between group 1 and all the other groups as well as between group 2 and group 4 (*p*‐values < .01). It is worth pointing out that all these results would have also been statistically significant when using Bonferroni correction. In group 2 vs. group 3, we computed a *p*‐value of .4649; the comparison of groups 3 and 4 yielded the result of *p* = .12499. Figure 1 shows the ferritin levels along with the medians for the respective groups, as well as the two different levels for the definition of ID (Achebe, WHO). Since values below 15 µg/l are not reported exactly, we were not able to show the interquartile range (IQR).

**TABLE 1:** Patients (counts) were divided into four groups, depending on gestational age at inclusion

Group	Weeks	Patients
1	11 + 0 to 14 + 6	115
2	24 + 0 to 32 + 6	89
3	33 + 0 to 37 + 6	51
4	38 + 0 to 41 + 6	170

**TABLE 2:** Medians for age, BMI, serum ferritin with respect to the different groups

	All	Group 1	Group 2	Group 3	Group 4
Age	31	31	32	30	31
BMI	23.3	22.4	24.35	24.4	23.4
Serum ferritin (μg/l)	22.7	38.7	23.2	18.1	16.5

**FIGURE 2:**
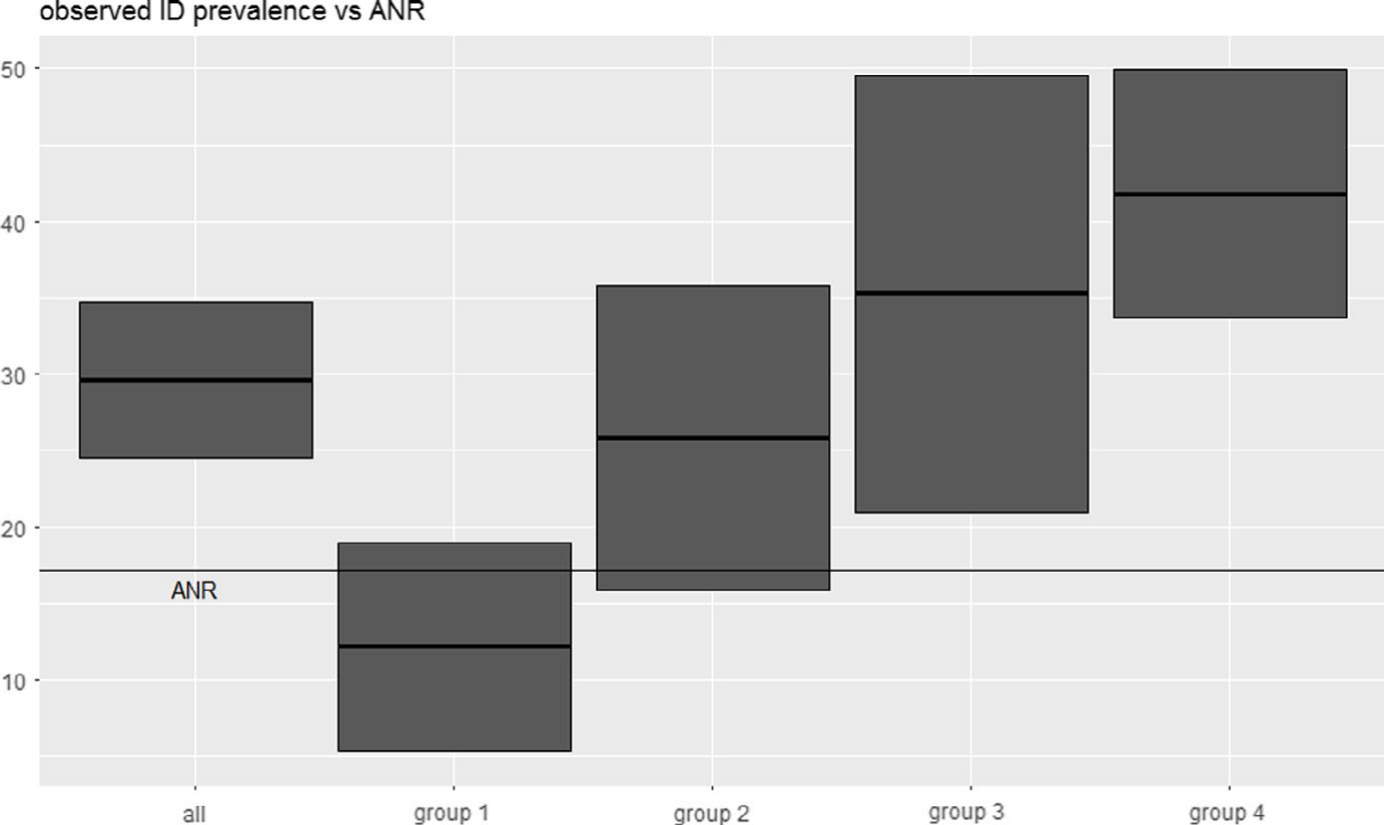
Boxplots (groups/observed prevalence) showing the rise of ID prevalence throughout the pregnancy with respect to the WHO’s definition of ID. The bold lines in the middle of the boxes show the observed values, while the bottom and top of the boxes represent the 95% CI. For comparison sake, we included a box based on the data of the entire cohort, as well as a line representing the ANR’s prediction

Twelve percent presented with ID during their first visit are lower than the ANR prediction (17.2%), and group 2 shows a rise of cases (about 1 in 4 patients tested positive for ID). In group 3, more than 1 in 3 patients were found to be affected by ID, and in group 4, we observed a prevalence of more than 40%. Overall, almost 30% of all patients were diagnosed with ID. Using the 95% confidence intervals, we expect at least 1 in 4 patients to develop ID over the course of the pregnancy and up to 50% among all patients with near‐term deliveries. With the exception for group 1, all these findings were statistically significant with *p*‐values < 0.01 (Figure 2, Table 3). Figure 2 shows the observed prevalence (bold lines) and the 95% CI (top and bottom of the box) for the respective groups; for comparison purpose, we included a line representing the ANR prediction. This tendency (the higher the gestational age the more likely ID develops) is also present in group 1. Using the WHO definition of ID, the prevalence for ID was 12% (gestational age of 11 + 0 to 14 + 6); at the second visit (2 months later), we again observe 12% of such cases (6 out of 49). However, considering that 14 patients were excluded from the second visit because they already were diagnosed with ID, there are now actually at least 20 out of 63 (31.7%) patients suffering from ID. Furthermore, an additional 30 had to be excluded from the second visit because iron supplement therapy was administered as well, due to their serum ferritin level being between 15 (including) and 30 (excluding). At the third visit, 3 out 15 (20%) presented with ID. Furthermore, 18 had to be excluded from this visit, again because of a serum ferritin level <30 μg/l. Overall with respect to the longitudinal arm, 23 out of 115 (20%) developed ID over the course of the pregnancy. Additionally, 58 presented with serum ferritin levels between 15 and 30 at either the first or second visit (50.44%) and 22 dropped out willfully (Table 4). We assume that a significant number of these patients would have also developed ID. This assumption is backed up by our above calculations for differences in the groups via Kruskal–Wallis, respectively, the pair‐wise Wilcoxon comparisons and the observation that median serum ferritin levels decline through the course of the pregnancy: 38.7 μg/l for group 1, 23.2 μg/l for group 2, 18.1 μg/l for group 3, and 16.5 μg/l for group 4 (median overall: 22.7 μg/l) (Table 2). However, since this point of view is a bit coarse, we also used Kendall's rank correlation (using all available data) to examine the relationship between the serum ferritin level and gestational age (in weeks). In order to tighten the conclusion, we added the Kendall's rank correlation.

**TABLE 3:** Observed prevalences for the four groups compared with the Austrian Nutrition Report's prediction via goodness of fit test and Confidence intervals

Groups	% iron deficiency	*p*‐Value^a^	Confidence interval 95%^b^
Group 1	12.17	.3243	5.3 – 19.1
Group 2	25.84	.007016	15.8 – 35.8
Group 3	35.29	9.385e−05	20.9 – 49.6
Group 4	41.76	<2.2e−16	33.6 – 49.9
All	29.65	1.049e−11	24.5 – 34.7

^a^ Chi‐square goodness of fit test.

^b^ Two‐sample test for equality of proportions with continuity correction.

**TABLE 4:** Longitudinal data concerning iron status for patients from group 1 (absolute frequencies)

Iron status (Serum ferritin μg/l)	Visit 1 (115 Patients)	Visit 2 (49 Patients)	Visit 3 (15 Pats)	Total
Iron deficiency				
<15	14	6	3	23
15–30	30	18	10	58
<30	44	24	13	81
Normal iron status				
>15	101	43	12	‐
>30	71	25	2	‐
Drop out/LoFU	‐	24	10	34

**FIGURE 3:**
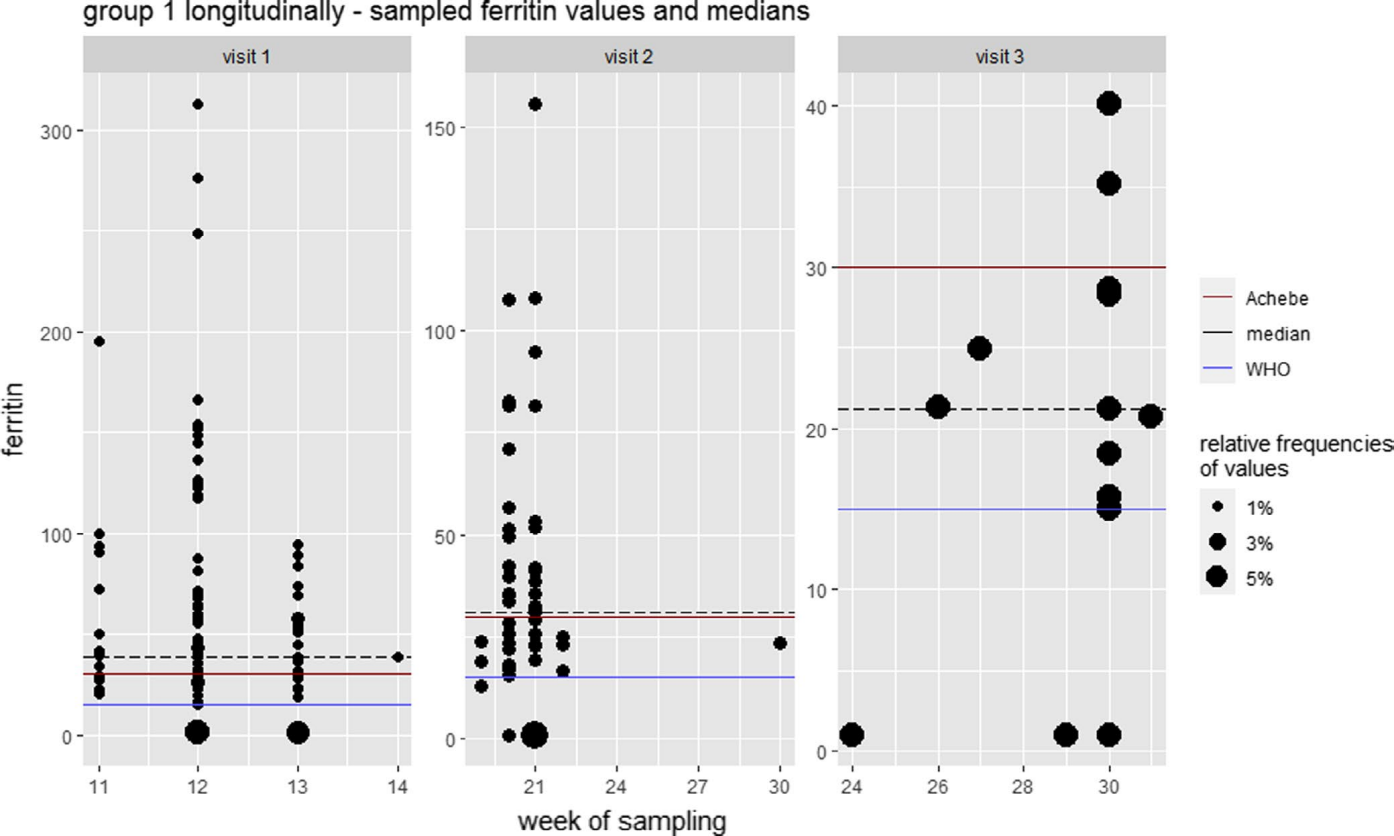
Scatter plot (gestational age in weeks at sampling/ferritin levels) detailing the distribution of ferritin levels at each visit. Each dot corresponds to one observed value in the respective week and its size scales with the number of samples with the respective value. As such, the size reflects relative frequencies of the sampled values per week on a visit level. For easy comparison, the median of the respective visit (black dashed line) was included, as well as the two different cutoff points for the definition of ID (red = ÖGGG, blue = WHO). For better readability, different scales were used for each visit

According to Achebe definition instead of WHO definition of ID (<30 μg/l serum ferritin vs. <15 μg/l serum ferritin), we are confronted with more than double the number of cases (for group 1, the number of cases rose from 13 to 42 (out of 115 patients); for groups 2 to 4, we observed an increase from 112 to 230 (out of 340 patients) cases. As a result about 1 in 3 patients presents with low serum ferritin levels already at the beginning of the pregnancy, and by the end of it, we observe a deficiency in 2/3 of all women (for 271 out of the 310 patients from groups 2–4, blood sampling was done within one week before delivery) (Figure 3).

## 4 DISCUSSION

To date, there are no data available on the prevalence of ID in pregnant women in Austria. The ANR is the only source for data about prevalence of ID in Austrian women in the reproductive age. Even when considering the divergent definitions of ID, the observed prevalence for patients of a gestational age of 24 + 0 or later deviate from ANR’s predictions substantially. In the longitudinal arm of the study, it could be shown that the prevalence increases significantly with the weeks of pregnancy. In group 4, we even found 41.76% of the patients to be suffering from ID. When using Achebe definition instead of WHO definition of ID, we are confronted with more than twice as many cases in total. The study participants were recruited by random selection. Self‐selection of study participants also takes place when health, language, and/or cultural barriers make participation difficult. The participating centers have a different number of births by year and are distributed throughout Austria, so that a representative cross‐section is achieved. This is further supported by the inclusion of patients in different weeks of gestation. We are aware that the selection of the pregnancy weeks for the group classification shows minimal deviations from the usual clinical relevance. However, this was necessary in order to achieve the optimal group size and does not affect the core statements of this study.

We have been looking at different cutoff points for diagnosing ID: <15 μg/l (according to WHO) and <30 μg/l (according to Achebe). However, ANR used yet another definition, namely serum ferritin below 10 µg/l. Since most of laboratories used by sites participating in this study report values below 15 μg/l simply as “< 15” (or in a similar fashion), we have not been able to directly compare our results to the one from ANR. As such, we diagnosed ID slightly more often than ANR would have. We also have to point out that in order to use the serum ferritin variable for statistical testing, we needed to choose a dummy value for “<15” (all these values were set to 1). To make sure this choice did not interfere with the results, we did another round of testing where the dummy values were acquired from a randomly generated sequence (uniform distribution with values between 1 and 14); while the p‐values changed slightly, we observed no change in (non)significance.

When checking for the correctness of an estimated prevalence of 17.2% for ID in Austria, we computed a precision ~0.035 for total group screening (*n* = 425); for groups 1 – 4 with samples sizes of 115, 89, 51 respectively 117, the precision drops to ~0.07, 0.08, 0.10, and 0.07 respectively (in each case at significance level 5% and 95% confidence interval) (Daniel, 1999). As such, we are confident in the findings for the group of all patients, as well as the results for groups 1 and 4, but we see the limitations of our calculations for the other groups, especially group 3. It should be noted that the time frame for inclusion for this subgroup was pre‐emptively extended to guarantee a group as large as possible (clinically speaking, one would assume inclusion for group 3 to start with 34 + 0 instead of 33 + 0). The pregnant woman was required to give birth within one week after taking the blood sample. It was originally intended to evaluate a possible association between ID and premature birth. Furthermore, we would have liked to include a discussion about IDA as well; however, since the prevalence for IDA is lower than the one for ID, we would have needed an even larger sample size. For the sake of completeness, 42 out of 425 patients represented with IDA according to WHO and an additional two when employing according to Achebe. When comparing our findings (9.88% of all patients presented with IDA) according to WHO (15.5% cases) via a chi‐square‐goodnes of fit test, we observe a statistically significant difference (*p*‐value < .01). Lastly, while statistical comparisons of the clinical parameters of the three subgroups (ID according to WHO, ID according to Achebe and patients with serum ferritin ≥30 μg/l) would have been of great interest to us, and the individual subgroup sizes have been deemed too low to allow for appropriate statistical testing. It is important to point out that ferritin is an acute phase reactant. When interpreting the ferritin values, however, it must always be taken into account that ferritin can be speciously normal or above normal. The determination of C‐reactive protein (CRP) and saturation of transferrin (TSAT) can support the correct interpretation.

## 5 CONCLUSION

The pregnancy care program “Mother‐Child‐Booklet” in Austria provides for two blood tests, namely up to 16 + 0 weeks of gestation and between 25 and 28 weeks of gestation. Colleagues who are convinced of possible adverse outcomes support the additional screening for ID. Due to the design of the study, it was not possible to show an association between ID and adverse outcomes. However, it is clear that ID can have a massive impact on quality of life. This alone justifies screening, as diagnosis and therapy are very simple. The aim should also be to avoid IDA at birth, in order to prevent higher maternal morbidity.

## CONFLICT OF INTEREST

HZ received lecture fees and a grant from Vifor Pharma Austria. FH, MM, JT, WD, CO, PK, VH, AR, EW, FW have nothing to declare.

## AUTHOR CONTRIBUTIONS

**Harald Zeisler:** Conceptualization (lead); Data curation (lead); Formal analysis (equal); Funding acquisition (lead); Investigation (lead); Methodology (lead); Project administration (lead); Writing‐original draft (lead); Writing‐review & editing (lead). **Wolf Dietrich:** Data curation (supporting); Writing‐review & editing (supporting). **Florian Heinzl:** Conceptualization (equal); Data curation (supporting); Formal analysis (lead); Methodology (equal); Writing‐original draft (equal); Writing‐review & editing (equal). **Philipp Klaritsch:** Data curation (supporting); Writing‐review & editing (supporting). **Victoria Humpel:** Data curation (supporting); Writing‐review & editing (supporting). **Manfred Mörtl:** Data curation (supporting); Writing‐review & editing (supporting). **Christian Obruca:** Data curation (supporting); Writing‐review & editing (supporting). **Friedrich Wimazal:** Conceptualization (supporting); Writing‐review & editing (supporting). **Angela Ramoni:** Data curation (supporting); Writing‐review & editing (supporting). **Johanna Tiechl:** Data curation (supporting); Writing‐review & editing (supporting). **Elisabeth Wenzel‐Schwarz:** Data curation (supporting); Writing‐review & editing (supporting).

## ETHICAL APPROVAL

The ethics committee of the Medical University of Vienna approved this study (EK 2010/2016).

## INFORMED CONSENT

Written Informed consent was obtained from all study participants.

## Data Availability

Data available on request from the authors.
